# Cost-effectiveness analysis of pneumococcal conjugate vaccine 13-valent in older adults in Colombia

**DOI:** 10.1186/1471-2334-14-172

**Published:** 2014-03-28

**Authors:** Jaime E Ordóñez, John J Orozco

**Affiliations:** 1 HEMO Group, Carrera 25 A # 1 A Sur-45, piso 5. Medellín, Colombia; 2 CES University, Calle 10 A # 22-04, Medellín, Colombia

**Keywords:** *Streptococcus pneumoniae*, Pneumococcal vaccines, Health economics, Middle aged, Aged, Colombia

## Abstract

**Background:**

Nowadays, there are two vaccination strategies in Colombia to prevent pneumococcal diseases in people over 50 years. Our aim is to estimate cost-effectiveness of pneumococcal conjugate vaccine 13-valent (PCV13) versus pneumococcal polysaccharide vaccine 23-valent (PPSV23) to prevent pneumococcal diseases and their related mortality in people over 50 years old in Colombia.

**Methods:**

A Markov model was developed with national data, including pneumococcal serotypes distribution in Colombia between 2005 and 2010. Vaccination of a cohort was simulated and a five year time horizon was assumed. Analysis was done from a perspective of a third party payer. Direct costs were provided by a national insurance company; sensitive univariate and probabilistic analysis were done for epidemiological and clinical effectiveness parameters and costs.

**Results:**

PCV13 avoids 3 560 deaths by pneumococcal infections versus PPSV23 and 4 255 deaths versus no vaccine. PCV13 prevents 79 633 cases by all-cause pneumonia versus PPSV23 and 81 468 cases versus no vaccine. Total costs (healthcare and vaccines costs) with PCV13 would be U.S. $ 97,587,113 cheaper than PPSV23 and it would save U.S. $ 145,196,578 versus no vaccine.

**Conclusion:**

PCV13 would be a cost-saving strategy in the context of a mass vaccination program in Colombia to people over 50 years old because it would reduce burden of disease and specific mortality by pneumococcal diseases, besides, it saves money versus PPSV23.

## Background

*Streptococcus pneumoniae* (pneumococcus) is a frequent cause of serious infectious diseases in children and adults. Pneumococcal infections may be invasive such as sepsis and meningitis [Invasive Pneumococcal Disease (IPD)], or non-invasive, such as pneumonia, the most common form of serious pneumococcal disease in adults [1]. According to the World Health Organization (WHO), in 2000 there were about 14.5 million cases of pneumococcal disease (invasive and noninvasive) [2].

Pneumococcal pneumonia is the most common bacterial cause of community acquired pneumonia (CAP) in adults [3]. Mortality rates for pneumococcal CAP are between 10% and 30% in adults and have remained constant over the past four decades [4-8].

Increased bacterial resistance [9], an increase in the prevalence of immunocompromised people, especially by HIV, immunosuppressive therapy in oncology, and transplantation medicine; as well, the increase in life expectancy that is accompanied by immune senescence processes likely contribute to sustained morbidity and mortality impact of this disease [10,11].

Given the burden of disease by pneumococcal diseases in worldwide, a prevention strategy with vaccination may be the most cost-effective approach to health systems. This is not a new topic, there are randomized clinical trials (RCTs) from 1940s [12,13], but it was just until 1977 that began the widespread use of this technology, when pneumococcal polysaccharide 14-valent vaccine (PPSV14) was authorized and later, in 1983, PPSV23 was launched. The latter is a vaccine made with 23 different capsular polysaccharide serotypes and has been indicated to prevent pneumococcal disease in children over two years old with risk factors and adults over 50 years old. International clinical guidelines generally recommend its use in adults over 65 years old, and for people under 65 years old, who have chronic comorbidities that put them at higher risk of disease [14].

In 2000, a pneumococcal vaccine with a different technology was launched, which conjugates pneumococcal serotypes with a diphtheria protein (CRM197). This one was the pneumococcal conjugate vaccine 7-valent (PCV7) that demonstrated its effectiveness [15-17] to prevent IPD, pneumonia and acute otitis media (AOM) caused by vaccine serotypes in children aged between 2 months and 9 years old. It has also been studied in adults, including patients with HIV [18]. Later, in 2006, a WHO expert group established that future pneumococcal conjugate vaccines may be evaluated on the basis of immunogenicity studies, such that efficacy studies with disease outcomes would not be necessary for regulatory approval. This statement was based on the PCV7 and PCV9 RCTs, that found an association between an antibody concentration > 0.35 mcg/ml of the serotypes contained in these vaccines and prevention of IPD by the respective serotype [19].

In 2010, a 13-valent pneumococcal conjugate vaccine (PCV13) was approved for prevention of IPD, pneumonia and AOM caused by serotypes included in the vaccine in children between 2 months and 5 years old. Later, in 2011, PCV13 was approved to use in people over 50 years to prevent pneumococcal diseases (IPD and pneumonia) caused by serotypes 1 3, 4, 5, 6A, 6B, 7 F, 9 V, 14, 18C, 19A, 19 F and 23 F.

Given that there are two technologies for prevention of pneumococcal diseases in people over 50 years old, the 23-valent polysaccharide vaccine and the 13-valent conjugate vaccine, the aim of this study was to estimate incremental cost-effectiveness ratio (ICER) of PCV13 versus PPSV23 and versus no vaccine in people over 50 years old in Colombia.

## Methods

### Model

**Figure 1:**
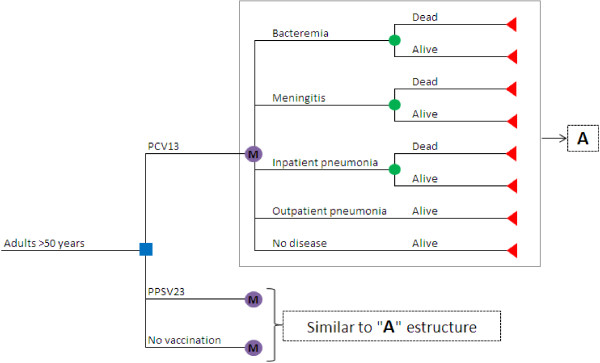
**Markov microsimulation model of pneumococcal diseases to determine the cost**-**effectiveness of vaccination with PCV13, PPSV23 or not vaccine, in people older than 50 years in Colombia.**

A cost-effectiveness analysis was performed using a Markov model with a first-order microsimulation to determine the risk of developing IPD and pneumonia in people over 50 years; the original model was developed by PAI (Policy Analysis Inc.) to Pfizer. The model simulates cohorts of individuals which represent the age and risk profile of the populations. Each individual’s risk was adjusted based on age and risk profile of individuals according to their medical history, such as chronic diseases or immunocompromised illnesses. No previous vaccination with PPSV23 was assumed. The model assumed that at the beginning, individuals would be vaccinated with PCV13 or PPSV23 or would not be vaccinated. Over the course of the simulations, individuals were at risk for four clinical outcomes: sepsis, meningitis, inpatient pneumonia and outpatient pneumonia. Mortality rates from IPD and pneumonia were considered to calculate deaths avoided. Risk reduction associated with the vaccines relied on the clinical presentation of diseases: IPD, inpatient pneumonia or outpatient pneumonia; as well as the vaccine, time since vaccination, age at time of diseases, and risk profile [20] (Figure 1) Further methodologic details can be referenced from application of the model to the US setting [20].

Costs of medical treatments were calculated based on the site of healthcare (inpatient or outpatient), age of patients, and their risk profile. To determine epidemiologic distribution of *S. pneumoniae* serotypes, we used the data of Regional Vaccine System (SIREVA II, by their acronym in Spanish), which reports serotypes of all isolates made in Colombia by IPD. For this purpose, we took the distribution of *S. pneumoniae* serotypes found in Colombia between 2005 and 2010 in people older than 50 years [21]. The model was calculated using a discount rate of 3% for both costs and effects, according to WHO recommendations [22].

Analysis was done from the perspective of a third-party payer in Colombia. Three alternatives were considered: no vaccination, vaccination with PCV13 or vaccination with PPSV23. The model horizon was five years and we assumed vaccination coverage of 70%. An incremental cost-effectiveness analysis was made among these three strategies in terms of avoiding cases and deaths for each disease and life-years gained (LYG).

The data used in this study are referenced in each item and are publically available in medical databases and official reports of Colombia, except hospitalization costs, which were provided by SURA, a national health insurance.

### Demographic and epidemiological parameters

The vaccinated cohort was calculated based on official data from a projection of population over 50 years in Colombia in 2012 (Table 1) [23]. The proportion of people in each age group with low, medium, or high risk for pneumococcal diseases or related complications was based on a study of pneumococcal disease burden in older adults in the United States (Table 2) [24]. The risk groups were classified as follows [25]:

•Low risk: immunocompetent people without chronic diseases.

•Medium risk: immunocompetent people with chronic diseases, such as cardiovascular, hepatic, lung illnesses or diabetes.

•High risk: immunocompromised people as a result of splenic dysfunction, malignancies, HIV, organ transplant or chronic renal failure.

**Table 1:** **Demographic and epidemiological parameters on the likelihood of developing pneumococcal diseases in people over 50 years in Colombia**, **2012**

**Parameter**	**Mean value**	**Data distribution**	**Reference**
Population distribution		No variation	[31]
50 – 64 years	5′963,690		
65 – 74 years	1′987,534		
75 – 79 years	641,029		
80 – 99 years	630,983		
Discount rate	3%	No variation	Assumption
Vaccination coverage	70%	Beta	Assumption
Epidemiological parameters:			
Incidence of bacteremia:		Beta	[24]
50-64 years	0.0667%		
≥ 65 years	0.181%		
Incidence of meningitis:		Beta	[24,25]
50-64 years	0.0046%		
≥ 65 years	0.0114%		
Incidence of all-cause pneumonia:		Beta	[24,25]
50-64 years	0.3281%		
≥ 65 years	2.1627%		
Bacteremia mortality:		Beta	[23,31]
50 – 64 years	3.5%		
65 – 74 years	5.7%		
75 – 84 years	11.4%		
85 – 99 years	27.5%		
Meningitis mortality:		Beta	[23,31]
50 – 64 years	26.7%		
65 – 74 years	19.5%		
75 – 84 years	37.4%		
85 – 99 years	40.0%		
Inpatient pneumonia mortality:		Beta	[23,31]
50 – 64 years	0.9%		
65 – 74 years	2.9%		
75 – 84 years	6.2%		
85 – 99 years	14.6%		
Costs (U.S. $)			
Inpatient pneumonia	Mean (SD)		
50 – 64 years	1,765 (4,005)	Gamma	Sura EPS (local data)
65 – 74 years	1,932 (4,721)		
75 – 79 years	2,688 (12,332)		
80 – 99 years	1,309 (3,355)		
Bacteremia			
50 – 64 years	10,434 (16,835)	Gamma	Sura EPS (local data)
65 – 74 years	8,495 (10,782)		
75 – 79 years	6,753 (10,380)		
80 – 99 years	4,849 (9,772)		
Meningitis			
50 – 64 years	7,789 (8,114)	Gamma	Sura EPS (local data)
65 – 74 years	7,783 (8,025)		
75 – 79 years	7,783 (8,025)		
80 – 99 years	7,783 (8,025)		

**Table 2:** **Distribution of people in each age group**, **with low**, **medium or high risk for pneumococcal diseases or their complications**[21]

**Age groups**	**Risk level**		
	**Low**	**Medium**	**High**
50 – 64 years	52.2%	36.6%	11.2%
65 – 74 years	41.7%	38.5%	19.8%
75 – 84 years	37.4%	36.9%	25.7%
85 – 99 years	36.6%	34.3%	29.1%

The model assumed that high-risk people who were vaccinated with PPSV23 will be revaccinated five years later [26].

We calculated the likelihood of falling ill with IPD, inpatient pneumonia or outpatient pneumonia based on two age groups of 50–64 years, and > 65 years. No literature that would identify the specific risk for additional age groups was found (Table 1) [27-30]. Mortality by IPD and pneumonia was calculated according to official data of general mortality by age groups in Colombia [31] and it was adjusted based on Koivula et al. [32].

Pneumococcal serotype distribution in adults older than 60 years between 2005 and 2010 in Colombia was based on reports of SIREVA II. Thus, proportion of *S. pneumoniae* serotypes circulating in Colombia covered by each vaccine was calculated, as well: PCV13 covers 64.6% and PPSV23 covers 76.9% [21].

### Vaccine effectiveness

#### IPD

Parameters of effectiveness of initial vaccination with PPSV23 in immunocompetent adults over 50 years and immunocompromised adults over 65 years were adapted from the approach by Smith et al., and it was adjusted for age, risk group, and time since vaccination [33]. As these data were taken from a panel of experts through Delphi methodology, sensitivity analysis and probabilistiva analysis were done [34], and these have been consistent with former results in the United Kingdom [35]. Effectiveness of initial vaccination with PPSV23 in immunocompromised adults between 50 and 64 years old in the year after vaccination was based on data from Shapiro et al. [34]. In the same way, we assumed a rate of decline in protection after vaccination [10]. It was assumed that the effectiveness of revaccination with PPSV23 was 75% of initial value (Table 3).

**Table 3:** PCV13 and PPSV23 effectiveness to prevent IPD in inpatient and outpatient pneumonia in adults over 50 years not previously vaccinated in the first year after vaccination

**Age groups (years)**	**PCV13**			**PPSV23**		
	**IPD **[14]**,**[33]**,**[34]	**Inpatient pneumonia **[28]	**Outpatient pneumonia **[10]	**IPD **[26]**,**[27]**,**[31]**,**[32]	**Inpatient pneumonia **[37]**-**[42]	**Outpatient pneumonia **[37]**-**[42]
50 – 64	88.9%	24.2%	5.6%	79.2%	0.0%	0.0%
65 – 74	81.5%	21.9%	5.1%	61.6%	0.0%	0.0%
75 – 79	75.7%	20.2%	4.7%	50.4%	0.0%	0.0%
80 – 99	70.3%	18.7%	4.3%	42.1%	0.0%	0.0%

*IPD*: Invasive Pneumococcal Disease.

Effectiveness of PCV13 for people over 50 years in the groups of low and moderate risk in the year after vaccination, was adapted from the effectiveness of PCV7 for children [15,35,36], assuming similar effectiveness against the six pneumococcal serotypes not included in PCV7. It was assumed a conjugate vaccine would have similar effectiveness as in children, but that this effectiveness would decline with increasing age after 50 due to immune-senescence. The decline in PCV13 effectiveness with age in people over 50 years was assumed as 50% of the corresponding rate of decline for PPSV23. Further for people in high-risk group, it was assumed PCV13 would have 78% of the corresponding age-specific effectiveness of adults with low and moderate risk [37]. It was assumed that effectiveness of both vaccines was constant across each of the serotypes contained in each vaccine, according to WHO recommendation of non-inferiority in the proportion of vaccine serotypes with antibody levels > 0.35 mcg/ml [38,39] (Table 3).

#### All cause-pneumonia

An effectiveness of zero (0) for PPSV23 against all-cause pneumonia was assumed; based on several studies that also have served as information source in other economic assessments [40-45]. For PCV13, effectiveness observed in children with radiological-confirmed pneumonia was assumed in inpatient pneumonia and it was adjusted for the effect of immune-senescence following a similar procedure as described with IPD [10]. For outpatient pneumonia, the starting point for effectiveness estimation was 6%, based on the percent reduction in children with pneumonia without radiological confirmation [11]. We assumed the same decline rate of effectiveness adjusted by age, used in patients over 50 years with IPD. In immunocompromised patients, it was assumed a PCV13 effectiveness of 65% less than immunocompetent adults of the same age, based on data of Klugman et al. [37] (Table 3).

### Economic parameters

Vaccine costs were taken from the Pan American Health Organization Revolving Fund prices; which lists values of different vaccines for the Americas in 2013; cost per dose of PCV13 is U.S. $ 15.84 and cost per dose of PPSV23 is U.S. $ 6.60 [46]. For administration cost for application of either vaccine, we assumed a value of U.S. $ 1. To calculate these values in American dollars, we assumed a value of $COP 1800 per U.S. $ 1 [47].

To determine the costs of treatment of sepsis, meningitis and pneumonia in adults over 50 years in Colombia, we took the information from a health insurance company with national presence and over 1.8 million members, which ensures the representativeness of Colombian population. Costs were determined based on the value actually paid by insurance companies to different hospitals and includes all services rendered during the stay including visits-hospital, diagnostic aids, antibiotics and other treatments necessary for recovery until discharge. These costs are based on national tariff manuals. In 2012, this insurance company reported a total of 48 cases of meningitis, 1 526 cases of pneumonia and 389 cases of sepsis. These costs were validated with data from a study in three hospitals in Bogota, Colombia, for a period of 18 months in 2011 [48]. The values were adjusted at 3.73%, which was the Consumer Price Index for 2011 reported by National Administrative Department of Statistics of Colombia (DANE by their acronym in Spanish) [23].

### Sensitivity analysis

Probabilistic and univariate sensitivity analyses were made for epidemiological parameters, effectiveness of interventions, and costs included in the model. The parameters that generated a higher level of uncertainty were identified and variance reported. For probabilistic sensitivity analysis a Monte Carlo simulation with a thousand iterations was performed, in order to evaluate each expected value in the distribution of costs and diseases likelihood for each strategy. In the base case, the probabilistic sensitivity analysis report both there were 7,5000,000 simulations and 1,000 trials.

We assumed a gamma distribution for medical costs avoided and the costs of vaccines, considering the kurtosis thereof. Alpha and beta parameters were calculated from standard deviations of the actual data. Results of Monte Carlo simulation show robustness of the model. A univariate sensitivity analysis was made with minimum and maximum values, with the aim to observe the sensitivity of ICER to change in the parameters of each variable.

### Cost-effectiveness analysis

A cost-effectiveness analysis was made to calculate the ICER of vaccines and number of avoided cases in relation to the four conditions of interest: sepsis, meningitis, inpatient pneumonia, and outpatient pneumonia. The calculation was made considering in numerator the costs difference of both alternatives and in denominator their effectiveness difference. Costs were result of the value of one dose of PCV13 or PPSV23, as well as the lower cost incurred on medical treatment for least number of cases of each disease.

Cost-effectiveness analysis of vaccines in relation to specific mortality from sepsis, meningitis and pneumonia and mortality added were made also. Finally, a cost-effectiveness analysis was made in relation to the LYG for each strategy.

## Results

Compared to no vaccination, use of PCV13 in adults over 50 is expected to prevent 49 857 cases of pneumococcal bacteremia; 3 135 of pneumococcal meningitis; 496 795 of inpatient pneumonia, 866 189 of outpatient pneumonia; and 33 980 deaths from pneumococcal infections, over 5 years. Moreover, total costs with the not vaccinating strategy were U.S. $ 145,196,578 greater than vaccinating with PCV13 (Table 4).

**Table 4:** **Cases and direct costs by pneumococcal infections vaccinating with PCV13**, **PPSV23**, **or not vaccine in people over 50 years in Colombia**

**Parameters**	**PCV13**	**PPSV23**	**No vaccination (NV)**	**PCV13**-**PPSV23**	**PCV13**-**NV**
Number of cases					
Bacteremia	34 934	39 809	49 857	−4 875 (−12.2%)	−14 923 (−29.9%)
Meningitis	2 306	2 557	3 135	−252 (−9.8%)	−830 (−26.5%)
Inpatient pneumonia	439 971	496 752	496 795	−56 781 (−11.4%)	−56 824 (−11.4%)
Outpatient pneumonia	841 546	864 397	866 189	−22 852 (−2.6%)	−24 644 (−2.8%)
Deaths by pneumococcal infections	29 726	33 285	33 980	−3 560 (−10.7%)	−4 255 (−12.5%)
LYG	11 377	2 290	0	9 087	11 377
Total costs (million)					
Medical costs	U.S. $ 1,286.2	U.S. $ 1,439.7	U.S. $ 1,534.9		
Vaccine costs	U.S. $ 103.6	U.S. $ 47.6	--		
Total	U.S. $ 1,389.8	U.S. $ 1,487.3	U.S. $ 1,534.9	U.S. $ -97.5	U.S. $ -145.1

Both vaccination strategies would prevent more deaths and IPD that no vaccination and PCV13 would reduce more than PPSV23. A mass vaccination program in Colombia for adults over 50 years with PCV13 would avoid 79 633 additional cases of pneumonia, both inpatient and outpatient, to those prevented with PPSV23. Furthermore, PCV13 would prevent and additional 5 127 cases of IPD and 3560 deaths by pneumococcal infections, more than PPSV23 (Table 4).

Total costs (medical costs and vaccination) were highest with the strategy of not vaccinating, and both PPSV23 and PCV13 were cost saving compared with no vaccination. However, vaccination with PCV13 saves U.S. $ 97 million versus PPSV23 (Table 4).

### Cost-effectiveness analysis

PCV13 is dominant over PPSV23 in terms of mortality avoided and LYG in adults over 50 years, because PCV13 avoids more deaths, generates more LYG, and healthcare costs expected would be lower than PPSV23. ICER indicates that PCV13 would be dominant over PPSV23, both in terms of illness and deaths averted by pneumococcal disease as well as LYG (Table 4).

### Sensitivity analysis

PCV13 would prevent 52.0% of the cases of pneumococcal bacteremia and meningitis and 12.6% of all-causes pneumonia versus no vaccination strategy, unlike PPSV23 that would prevent 26.9% and 6.2%, respectively (Table 4).

**Figure 2:**
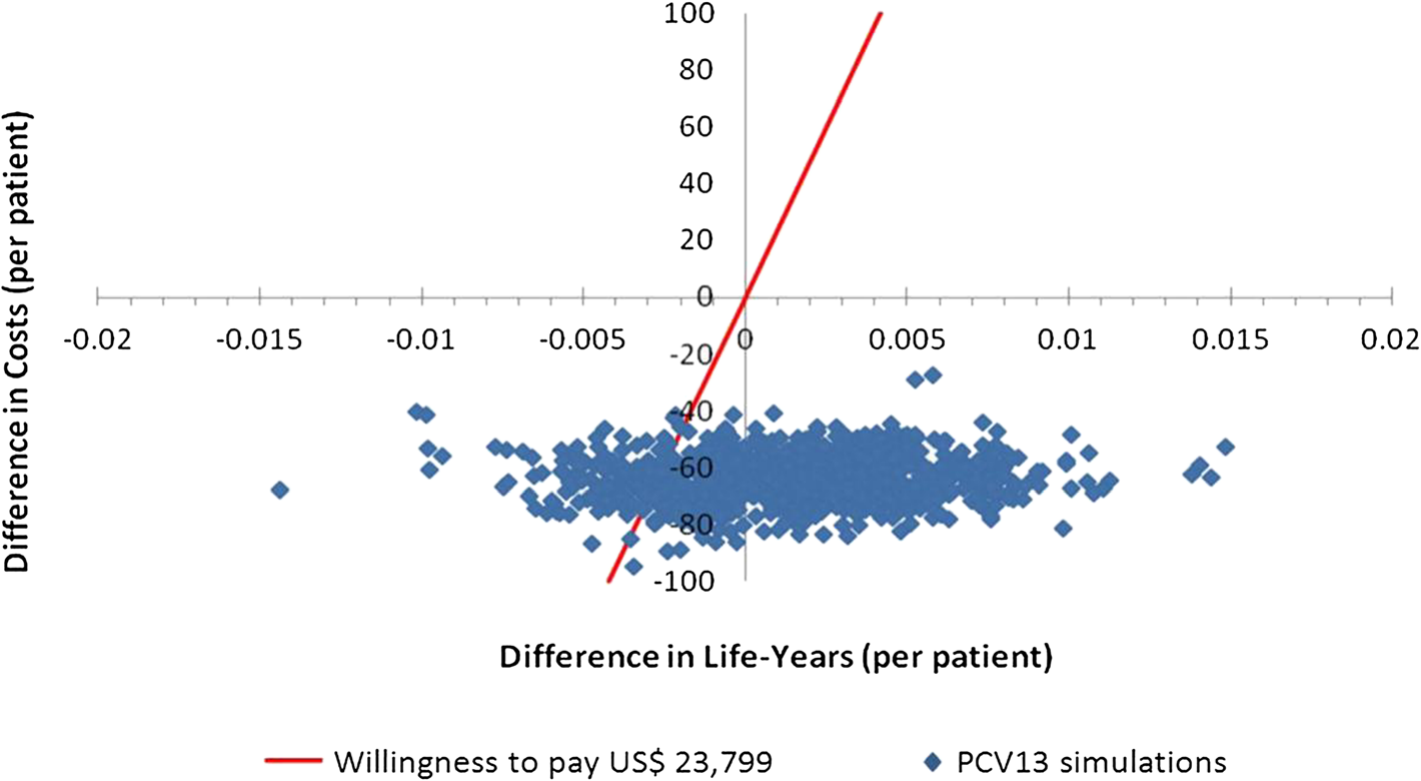
**Probabilistic sensitivity analysis**, **which plotted life years gained versus costs for PCV13 and PPSV23 in a cohort of people over 50 years in Colombia**, **2012.**

In the probabilistic sensitivity analysis, among one thousand simulations of the model, PCV13 had a lower cost than PPSV23 in 100% of cases, there was more LYG in 64% of cases and it was cost-effective in 87% of cases, taking into account a willingness to pay of 3 GPD (U.S. $ 23,799) [49] (Figure 2).

**Figure 3:**
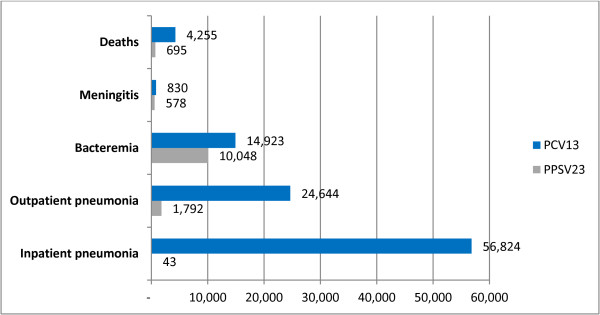
**Number of cases avoided with PCV13 and PPSV23 compared to no vaccination in a cohort of people over 50 years in Colombia, 2012.**

In the same way, in cost effectiveness plane, PCV13 is dominant over PPSV23 and no vaccination, which is due to be a strategy with greater clinical effectiveness and lower costs (Figure 3).

## Discussion

This cost-effectiveness analysis has identified both clinical and economic outcomes of the alternatives that exist in the Colombian market to prevent pneumococcal diseases in adults over 50 years: PCV13 and PPSV23. From the perspective of a third-party payer, both strategies are dominant versus non-vaccination, in terms of avoided diseases and deaths, ICER, and LYG. In addition, PCV13 was cost-saving, and is below the threshold of 1 per capita gross domestic product Colombian in 2012 (estimated at U.S. $ 7.933) [49], according to WHO recommendations [50].

Since 2009, several Colombian cities are including PPSV23 within their immunization plan. In 2011, PCV13 received health registration for use in people over 50 years to prevent pneumococcal diseases. Results of this cost-effectiveness analysis are intended to generate information to make decisions regarding the choice of two options that are on the market.

The results of this cost-effectiveness analysis are similar to those reported recently by Smith et al. and Weycker et al. in the US, both of which concluded that PCV13 in adults over 50 years of age is a better strategy than PPSV23 in terms of cost-effectiveness, to prevent pneumococcal infections in adults in the United States [24,51].

Although this cost-effectiveness analysis only considered the use of one dose of both, PCV13 or PPSV23 because the time horizon was five years, it is important to note that if the time horizon would have been the life expectancy, two doses of PPSV23 should be used for people at high risk, according to its manufacturers.

In the other hand, a meta-analysis published by Moberley et al. recommends PPSV23 versus placebo to prevent pneumococcal diseases [52]. RCTs used in that meta-analysis and published between 1980 and 2006 did not found any evidence about PPSV23 as protective vaccine to avoid pneumonia [40,41,53-58]. It means that the conclusion of Moberley et al. is based on only two RCTs: Kaufman (1948) [13] and Riley (1977) [59]. It is important to note that these RCTs [13,59] did not identified infection by *S. pneumoniae* but *Diplococcus pneumoniae*, which is a different microorganism.

Clinical effectiveness of pneumococcal conjugate vaccines for IPD in adults is judged by non-inferiority of antibody response compared with PPSV23. Jackson et al. showed that in people aged 70 years and older, opsonophagocytic activity titers were significantly greater in the PCV13 group versus PPSV23 group for 10 of the 12 serotypes common to both vaccines and to serotype 6A which is only in PCV13. Responses were non-inferior for the other 2 common serotypes [60]. Much effectiveness, particularly in pneumonia, is inferred based on PCV13 being a conjugated vaccine and eliciting a T-cell dependent response. This is supported by effectiveness in children reducing pneumonia and nasopharyngeal carriage, in fact, Cappuy et al. demonstrated that 2 to 4 years following PCV7 introduction, predominant pneumococcus serotypes carried in children with community-acquired pneumonia were non PCV7 serotypes [61].

A main strength of this study is that it has local information, which helps determine the epidemiological parameters for diseases of interest in Colombia [31]. Also, medical costs of treatments are based on local data [46] and are not supposed on studies done in other countries with different economic realities. In the same way, to adjust efficacy of both vaccines based on pneumococcal serotypes circulating in the country between 2005 and 2010, allows having an updated analysis [21]. So, by having local and updated economic and epidemiological information, we can develop a robust economic model, that allows to national and regional health authorities assess their vaccination plans and take decisions.

The biggest limitation of this study is that there is not effectiveness data for PCV13 in this population. However, the advantages of conjugation are expected to enhance effectiveness against mucosal disease, such as pneumonia, increase immune memory compared to plain polysaccharide vaccine, and improve effectiveness in high risk populations such as immunocompromised. In a study of PCV7 versus placebo in HIV patients, French et al. found a vaccine efficacy of 74% (95% CI: 30% - 90%) to prevent pneumococcal diseases [18]. In other study, Klugman et al. found that vaccination with PCV9 reduced the rates of radiologically confirmed pneumonia, and PCV9 also reduced the incidence of vaccine-serotype and antibiotic-resistant IPD among children with and those without HIV infection [37].

Another weakness of this study, is that there is limited information comparing clinical outcomes of PPSV23 and PCV13 in adults to prevent IPD, which are the most lethal diseases caused by pneumococcus. In the same way, by taking data of disease prevalence from an official source, it could be some underreporting, because quality of this kind of information depends that medical practitioners use the correct codes to identify diagnosis.

This analysis did not evaluate after-effects of any of the diseases, why not were estimated years of disability-adjusted life. But it is noteworthy that in terms of prevention of death from all causes, PCV13 was dominant over PPSV23.

## Conclusion

The results of this study indicate that PCV13 and PPSV23 are better alternatives in terms of cost-effectiveness compared with no vaccination. PCV13 is dominant over PPSV23, since PCV13 prevents more deaths by pneumococcal diseases, generates more LYG, and costs are expected to be lower than PPSV23 in adults over 50 years. Implementation of a vaccination program for adults older than 50 years in Colombia with PCV13 would decrease morbidity and mortality by pneumococcal diseases and would be a cost-saving strategy.

## Competing interests

To Sura EPS, a Colombian company health insurance, which provided the data for hospitalization costs.

## Authors’ contributions

JEO reviewed the scientific literature and identified local epidemiological data. JJO adapted economic model developed by Policy Analysis Inc. Both authors read and approved the final manuscript.

## Pre-publication history

The pre-publication history for this paper can be accessed here:

http://www.biomedcentral.com/1471-2334/14/172/prepub
